# Prenatal diagnosis and outcomes of fetal cardiac tumors: a 10-year single-center study of 81 cases

**DOI:** 10.3389/fmed.2026.1894872

**Published:** 2026-08-13

**Authors:** Longzhuang Peng, Yangqing Zuo, Jinwen Chen, Zongjie Weng, Qiumei Wu, Qiong Huang, Wen Ling

**Affiliations:** Department of Medical Ultrasonics, Fujian Maternity and Child Health Hospital, College of Clinical Medicine for Obstetrics and Gynecology and Pediatrics, Fujian Medical University, Fuzhou, China

**Keywords:** echocardiography, fetal cardiac tumors, prenatal diagnosis, prognosis, tuberous sclerosis complex

## Abstract

**Objective:**

This study aimed to comprehensively analyze the prenatal phenotype, genetic basis, and postnatal trajectory of a large cohort of fetuses with fetal cardiac tumors (FCTs).

**Methods:**

This retrospective study included 81 fetuses diagnosed with FCTs at our center between 2014 and 2024. We reviewed prenatal ultrasound and MRI findings, genetic testing results, and postnatal follow-up data, extending to a minimum follow-up period of 1 year. Tumor characteristics, complications, and predictors of tuberous sclerosis complex (TSC) and adverse outcomes were analyzed.

**Results:**

Among the 81 cases FCTs, cardiac rhabdomyoma (CR) was the most common tumor (66 cases, 81.5%), followed by fibroma (*n* = 5, 6.2%), myxoma (*n* = 3, 3.7%), hemangioma (*n* = 1, 1.2%), teratoma (*n* = 1, 1.2%), and unclassified tumors (*n* = 5, 6.2%). Complications occurred in 30.9% of cases and were associated with tumor volume and location rather than tumor multiplicity. TSC was identified in 70.0% (42/60) of fetuses with CR. Tumor multiplicity was significantly associated with TSC (78.7% for multiple vs. 38.5% for solitary lesions, *p* = 0.013), whereas maximum tumor volume did not differ significantly between TSC-associated and isolated CR (*p* = 0.646). Cranial MRI increased prenatal TSC detection by 23% compared with ultrasound alone (69.2% vs. 46.2%). Among the 48 live-born infants (live birth rate 59.3%), 22 cases showed tumor regression or disappearance during follow-up, all of which were CRs. One case each of CR, fibroma, and an unclassified tumor showed gradual enlargement postnatally. Six cases underwent surgical resection, and the remaining tumors remained stable. Among children with TSC, 72.7% (16/22) developed epilepsy, and 36.4% (8/22) had neurodevelopmental disorders, whereas all TSC-negative children had normal neurodevelopment.

**Conclusion:**

Tumor multiplicity, rather than size, was more strongly associated with TSC in fetal CR. Ultrasound combined with MRI improved the prenatal detection rate of fetal TSC. In the absence of hemodynamic compromise and TSC, FCTs had a favorable prognosis. These findings support a management algorithm based on tumor type, multiplicity, tumor size/location, and genetic testing.

## Introduction

Fetal cardiac tumors (FCTs) are a group of rare congenital abnormalities with a variable prognosis, with an estimated prevalence of 0.024–0.25% (1, 2). Histologically diverse, CR is the most common type, accounting for 60–90% of cases, followed by teratoma, fibroma, hemangioma, and myxoma (2). Although the majority of FCTs are benign with generally favorable tumor-related outcomes (3), some tumors can cause serious complications, including valve regurgitation, outflow tract obstruction, arrhythmias, heart failure, and even intrauterine death (2, 4). Furthermore, FCTs frequently represent the initial manifestation of certain genetic syndromes (3), particularly because of the strong association between CR and TSC (5, 6). TSC can lead to epilepsy, intellectual disability, and multisystem involvement (7, 8), which significantly affects long-term prognosis.

Prenatal echocardiography is the first-line modality for diagnosing FCTs (9), enabling the real-time assessment of tumor number, size, location, morphology, and hemodynamic impact. However, ultrasound has limitations in distinguishing among different pathological types of FCTs and has limited sensitivity for detecting TSC-associated intracranial lesions (10). Fetal brain MRI, with its superior soft-tissue resolution, serves as an important complementary tool (11). Additionally, molecular testing for the TSC1 and TSC2 genes provides the diagnostic gold standard (10, 12).

Despite advances in diagnostic techniques, precise prenatal diagnosis and prognostic assessment of FCTs remain challenging. Previous studies have shown that a considerable proportion of FCT-affected pregnancies are terminated due to a lack of definitive prenatal diagnosis (13), while affected children may experience adverse long-term outcomes without timely postnatal intervention (14). Large-scale systematic studies on FCTs remain limited, and data on natural history, complication profiles, genetic associations, and perinatal management strategies remain insufficient. This study presents a systematic retrospective analysis of 81 FCT cases from a single regional prenatal diagnosis center over a decade, comprehensively summarizing the pathological spectrum, prenatal imaging characteristics, genetic associations, complications, and pregnancy outcomes.

## Methods

### Study population

This retrospective study initially identified 86 fetuses diagnosed with FCTs at Fujian Maternity and Child Health Hospital between June 2014 and June 2024, from a cohort of 139,385 pregnant women who underwent systematic prenatal ultrasound examinations. After excluding five cases (including one case that was postnatally confirmed as an intracardiac thrombus), 81 fetuses were finally included. The inclusion criteria were as follows: prenatal ultrasound diagnosis of FCTs, complete ultrasonographic records, available pregnancy outcomes, and at least 1 year of postnatal follow-up. The exclusion criteria were as follows: incomplete data, loss to follow-up, or postnatal confirmation of non-neoplastic lesions. The study was approved by the hospital’s Ethics Committee (No. 2024KY259).

### Prenatal multidisciplinary diagnosis

Ultrasound examinations were performed using GE Voluson E8 or E10 systems (2–9 MHz convex transducer) following International Society of Ultrasound in Obstetrics and Gynecology (ISUOG) guidelines (15, 16). For each cardiac tumor, a comprehensive assessment was performed, documenting its location, size (with volume calculated as length × width × height × 0.523), number (solitary vs. multiple, with multiple defined as ≥2 lesions), morphology, echogenicity, vascularity, and relationship to the adjacent myocardium. Concurrently, a thorough fetal survey was conducted to screen for associated intracardiac or extracardiac anomalies and complications. The fetal brain was systematically examined for intracranial lesions, particularly subependymal nodules and cortical tubers.

Fetal brain MRI was recommended for suspected CR but was performed only after obtaining parental consent. Examinations were performed on a 1.5-T scanner (GE Healthcare, Chicago, IL, USA) using single-shot fast spin-echo (SSFSE), fast image employing steady-state acquisition (FIESTA), diffusion-weighted imaging (DWI), and T1 fat-suppressed sequences. Images were reviewed by a radiologist blinded to ultrasound findings, with particular attention paid to subependymal nodules and cortical tubers.

Genetic testing for the TSC1 and TSC2 genes was offered following genetic counseling and was performed via amniocentesis or cordocentesis only after parental consent was obtained. Next-generation sequencing (covering all coding exons with a sequencing depth of >100×) was used. Variants were interpreted according to the American College of Medical Genetics and Genomics / Association for Molecular Pathology (ACMG/AMP) guidelines (17).

### Follow-up and outcome assessment

Continuing pregnancies were monitored by ultrasound every 2–4 weeks. For pregnancies ending in termination, fetal autopsy was performed after obtaining informed consent. Live-born infants underwent echocardiography within the first week after birth. TSC-suspected newborns underwent skin and eye examinations, brain MRI, abdominal ultrasound, and EEG. Postnatal follow-up at 6, 12, and 24 months assessed tumor changes, surgical interventions, seizures, and neurodevelopment using the Gesell Developmental Scale.

### Tumor classification

**Figure 1:**
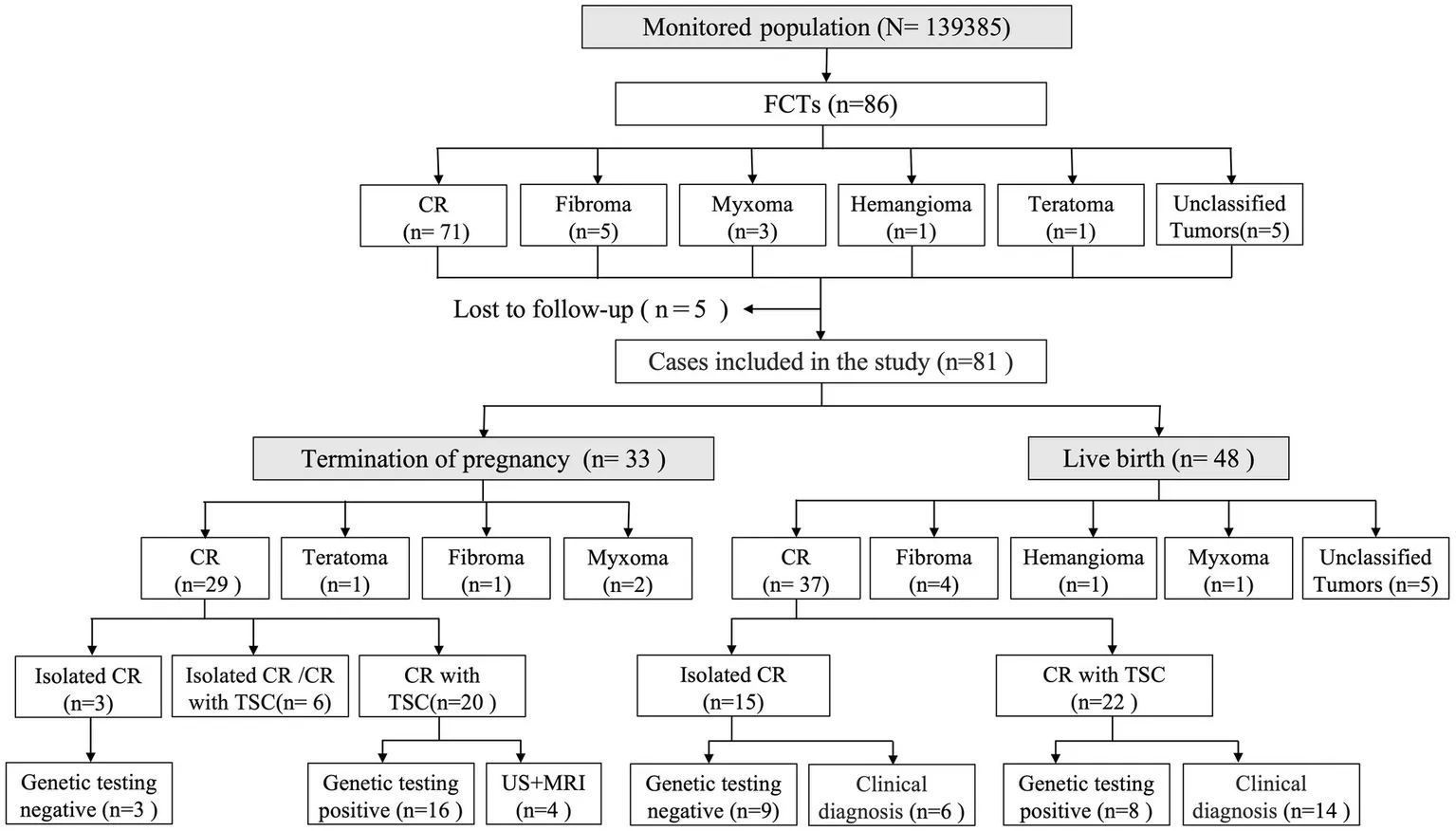
Distribution of pathological types and pregnancy outcomes of FCTs in this study.

Pathological diagnosis was considered the gold standard. TSC was diagnosed according to the international consensus criteria (18). In the absence of prenatal genetic testing, the diagnosis of TSC was made prenatally based on the identification of cardiac lesions suggestive of CR on ultrasound, together with concurrent characteristic intracranial abnormalities detected on both ultrasound and MRI. Cases with positive TSC genetic test results were classified as CR. For cases without pathological or genetic results, a multidisciplinary team established the diagnosis based on imaging findings, postnatal course, and clinical presentation. Tumors without definitive classification were categorized as unclassified. The classification of FCTs and the specific modalities applied for TSC diagnosis are comprehensively summarized in Figure 1.

### Statistical analysis

All statistical analyses were performed using SPSS 27.0. Categorical variables were described as frequencies and percentages, and normally distributed continuous variables were expressed as the mean ± standard deviation (SD). Non-normally distributed tumor volume data were compared using the Mann–Whitney U test. The chi-squared test or Fisher’s exact test was used for categorical intergroup comparisons, while analysis of covariance (ANCOVA) was performed to adjust for gestational age and tumor number to reduce potential confounding. We calculated odds ratios (ORs) with 95% confidence intervals (CIs) and Cramer’s V to assess association strength. A *p*-value of < 0.05 indicated statistical significance.

## Results

### General characteristics

Among 139,385 screened pregnancies, 81 FCTs were identified (a detection rate of 0.062%). Maternal age averaged 27.95 ± 3.88 years (range, 19–42 years), and gestational age at diagnosis averaged 25.86 ± 4.07 weeks (range, 18–39 weeks), with 50.6% of diagnoses occurring after 30 weeks of gestation. The distribution of pathological types is shown in Figure 1. CR was the most common diagnosis, accounting for 66 cases (81.5%). Fibroma was identified in five cases (6.2%) and myxoma in three cases (3.7%). Rare tumor types included hemangioma (one case, 1.2%) and teratoma (one case, 1.2%).

Multiple tumors were present in 53 cases (65.4%), and all non-CR tumors were solitary. The mean tumor volume was 2.63 ± 7.82 cm^3^ (range: 0.02–61.96 cm^3^). The most common locations of FCTs were the left ventricular free wall (54 cases, 66.7%), the right ventricular free wall (50 cases, 61.7%), and the interventricular septum (26 cases, 32.1%). The prenatal characteristics of FCTs are summarized in Table 1.

**Table 1:** Prenatal characteristics of FCTs.

Tumor type	Total (*n*)	GA at diagnosis (*n*)	Location (*n*)	Number of tumors (*n*)	Largest volumes (cm^3^), mean ± SD (range)	Combined malformations (*n*)	Complication rate, *n* (%)	Complications (*n*)
CR	66	18–24 w (5) 24–30 w (28) 30–36 w (31) >36 w (2)	LV (49) RV (47) IVS (26) RA (12) LA (2)	Single (13) Multiple (53)	1.15 ± 3.22 (0.02–16.45)	ASD (1) VSD (1) Choroid plexus cysts (1) Cyst of the cord (1) Plantar flexion of the toes (1) Bilateral foot inversion (1) Cleft lip and palate (1) Asymmetric cry syndrome (1)	17 (25.8)	Outflow obstruction (6) TR (3) Arrhythmia (3) MR (2) PE (2) HA/CA > 0.45 (2) Heart failure (2) Polyhydramnios (1) Hyposarca (1)
Fibroma	5	18–24 w (1) 24–30 w (1) 30–36 w (3)	LV (4) RV (1)	Single (5) Multiple (0)	23.29 ± 23.41 (3.36–61.96) ^a^	0	3 (60.0)	PE (3) MR (1) Arrhythmia (1)
Myxoma	3	18–24 w (1) 24–30 w (1) 30–36 w (1)	LA (1) AS (1) BV (1)	Single (3) Multiple (0)	0.48 ± 0.33 (0.16–0.81)	VSD (1)	3 (100)	MR (1) PE (3) HA/CA > 0.45 (1) Heart failure (1) Arrhythmia (1)
Hemangioma	1	>36 w (1)	RA (1)	Single (1) Multiple (0)	3.63 (single value)	0	1 (100.0)	PE (1)
Teratoma	1	24–30 w (1)	AD (1)	Single (1) Multiple (0)	6.81 (single value)	Right coronary artery right atrial fistula (1)	1 (100.0)	PE (1)
Unclassified	5	18–24 w (1) 24–30 w (1) 30–36 w (2) >36 w (1)	LV (2) RV (2) AS (1)	Single (5) Multiple (0)	0.34 ± 0.36 (0.04–0.95)	0	0 (0.0)	0

^a^Hemangioma and teratoma (*n* = 1 each) were excluded from statistical comparisons. The mean volume of fibromas was significantly greater than that of the other three tumour groups (*p* < 0.001), while no significant intergroup differences were observed among the remaining three groups. GA, gestational age; LA, left atrium; RV, right ventricle; IVS, interventricular septum; RA, right atrium; LV, left ventricle; AS, atrial septum; BV, bicuspid valve; AD, auricula dextra; ASD, atrial septal defect; VSD, ventricular septal defect; MR, mitral regurgitation; TR, tricuspid regurgitation; PE, pericardial effusion; HA/CA, heart area/chest area.

### Complications of FCTs and tumor changes after birth

Complications occurred in 25 fetuses (30.9%); the spectrum of complications included pericardial effusion (10 cases, 40.0%), valve regurgitation (8 cases, 32.0%), outflow tract obstruction (6 cases, 24.0%), cardiac arrhythmia (5 cases, 20.0%), and an increased cardiothoracic ratio (3 cases, 12.0%). There was no significant difference in complication rates between solitary and multiple tumors (35.7% vs. 28.3%, *p* = 0.456). However, tumor volume was significantly larger in the complication group (6.50 ± 12.76 vs. 0.91 ± 3.04 cm^3^, *p* < 0.001). All six cases of outflow tract obstruction resulted from tumors located in the outflow tract.

Among the 48 live-born infants followed for at least 1 year (median follow-up: 48 months, range: 12–97 months), lesion regression or disappearance was observed in 22 cases (45.8%), all of which were CR. Tumor enlargement during follow-up occurred in one case of CR, one case of fibroma, and one case of an unclassified tumor. The remaining tumors that did not undergo surgical treatment remained stable. Pregnancy outcomes of FCTs are presented in Table 2.

**Table 2:** Pregnancy outcomes of the FCTs.

Tumor type	Total (*n*)	TOP, *n*	Live birth, *n* (%)	Postpartum tumor change	Surgery, *n*
CR	66	29	37 (56.1)	Enlarged (1) Stable (14) Reduced (8) Disappeared (14)	1
Fibroma	5	1	4 (80.0)	Enlarged (1) Stable (1) Postnatal surgery (2)	3
Myxoma	3	2	1 (33.3)	Postnatal surgery (1)	1
Hemangioma	1	0	1 (100.0)	Postnatal surgery (1)	1
Teratoma	1	1	0 (0)	/	/
Unclassified	5	0	5 (100.0)	Enlarged (1) Stable (4)	0

TOP, termination of pregnancy.

### Analysis for various types of FCTs

#### CR

**Figure 2:**
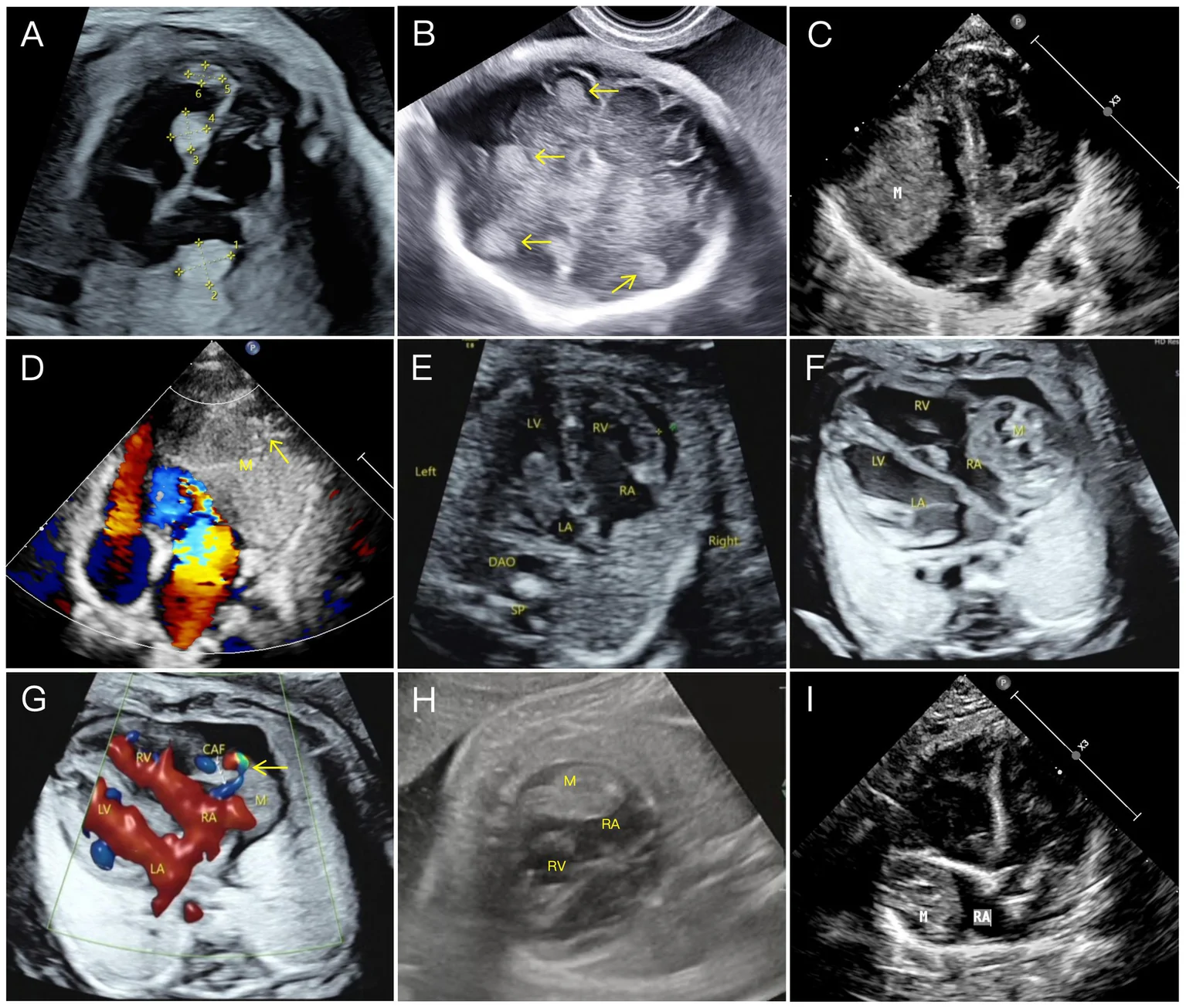
Sonographic features of various cardiac tumors. **(A)** At 31 + 3 weeks, the fetal heart shows multiple strongly echogenic CRs protruding into the cardiac chambers. **(B)** Cranial ultrasound of the fetus in **(A)** reveals multiple parenchymal brain lesions (arrows), suggestive of TSC. **(C)** A broad-based isoechoic fibroma extending across the left atrium and the basal portion of the left ventricle. **(D)** Fibroma involving the interventricular septum, apex, and lower lateral wall of the left ventricle, with heterogeneous internal echotexture and visible calcifications (arrow). **(E)** At 22 + 2 weeks, a myxoma was attached to the annulus of the anterior leaflet of the mitral valve, exhibiting a loose internal echo pattern. **(F)** At 29 + 1 weeks, a teratoma arising from the right atrial appendage and protruding into the pericardial cavity, presenting as a cystic-solid mass with calcification, accompanied by pericardial effusion. **(G)** The same case as in **(F)**, complicated by a right coronary artery–right atrial fistula (arrow). **(H)** At 39 + 3 weeks, a right atrial hemangioma presenting as a slightly hyperechoic mass with small anechoic areas and a broad base. **(I)** At 45 days after birth, the hemangioma has enlarged and exhibits a spongiform appearance.

Among the 66 CR cases, the mean gestational age at detection was 29.37 ± 4.57 weeks. The mean tumor volume was 1.15 ± 3.22 cm^3^. Multiple tumors were present in 80.3% (53/66) of cases. The most common locations were the left ventricular free wall (49 cases, 74.2%), the right ventricular free wall (47 cases, 71.2%), and the interventricular septum (26 cases, 39.4%). On ultrasound images, CR typically appeared as well-circumscribed, homogeneous, hyperechoic nodules protruding into the cardiac chambers (Figures 2A,B). Complications occurred in 17 cases (25.8%), mainly including outflow tract obstruction (6 cases), valve regurgitation (5 cases), and arrhythmia (3 cases). The three arrhythmias were sinus bradycardia, non-conducted premature atrial contractions, and supraventricular tachycardia; two resolved postnatally.

Fetal brain MRI was performed in 36 cases (9 with solitary tumors and 27 with multiple tumors), while the remaining 30 families declined the examination. There were no significant differences in gestational age, tumor number, or tumor size between fetuses who underwent MRI and those who did not (all *p* > 0.05). Among the 36 MRI-examined cases, 26 were ultimately diagnosed with TSC. Ultrasound alone detected intracranial lesions in 12 cases (46.2%, 95% CI: 26.59–66.67) and MRI detected lesions in 17 cases (65.4%, 95% CI: 44.33–82.80). The combined detection rate was 69.2% (18/26, 95% CI: 44.33–82.80). MRI-detected lesions were predominantly subependymal nodules, which were difficult to identify on ultrasound examinations.

After excluding six patients with unclear TSC status, definite TSC was identified in 70.0% of CR cases (42/60, 95% CI: 56.1–81.3). Multiple tumors were present in 88.1% (37/42) of patients with TSC-related CR vs. 55.6% (10/18) of patients with isolated CR (*p* = 0.013). No significant intergroup difference in maximum tumor volume was found between patients with TSC-related CR and those with isolated CR (0.85 ± 1.06 cm^3^ vs. 1.61 ± 3.03 cm^3^, *p* = 0.646; Table 3). ANCOVA adjusted for gestational age and tumor number further confirmed the absence of significant intergroup differences in tumor volume (*F* = 1.662, *p* = 0.203).

**Table 3:** Comparison of clinical and imaging features between isolated CR and CR with TSC.

Variable	Isolated CR	CR with TSC	Test statistic/*p*-value	Effect size (95% confidence interval)
Maximum tumor volume, cm^3^	1.61 ± 3.03 Median: 0.295 (0.12, 1.86)	0.85 ± 1.06 Median: 0.302 (0.21, 0.97)	*U* = 349, *p* = 0.646^a^	Median difference = −0.006 cm^3^ (−0.321–0.925)
Number of lesions, *n*/*N* (%)				
Single	8/18 (44.4)	5/42 (11.9)	χ^2^ = 7.86, *p* = 0.013	OR = 5.92 (1.58–22.11)
Multiple	10/18 (55.6)	37/42 (88.1)		
Postpartum changes in lesion size, *n*/*N* (%)				
Enlarged	1/15 (6.7)	0/22 (0)	χ^2^ = 2.40, *p* = 0.195	Cramer’s V = 0.255 (−0.078–0.512) ^b^
Stable	4/15 (26.7)	10/22 (45.4)		
Reduced	2/15 (13.3)	6/22 (27.3)		
Resolved	8/15 (53.3)	6/22 (27.3)		

^a^Maximum tumor volume exhibited non-normal distribution in both groups; hence, a non-parametric Mann–Whitney U test was applied for intergroup comparisons. ^b^Unordered four-category postnatal lesion evolution was assessed using Pearson’s *χ*^2^ test, and Cramer’s V was reported for effect size. OR, odds ratio; CI, confidence interval.

Of the 29 terminated pregnancies, 20 had a prenatal diagnosis of TSC, 2 were terminated for fetal supraventricular tachycardia and heart failure, respectively, and the remaining 7 were terminated at parental discretion. Among the 37 live-born infants with CR (median follow-up of 48 months, range: 14–97 months; 6 patients with follow-up <24 months, including five TSC cases), 22 presented with TSC and 15 with isolated CR. Twenty-two (59.5%) exhibited spontaneous tumor regression during follow-up. There was no significant difference in postnatal tumor change patterns between the two groups (*p* = 0.195) (Table 3). Neurological outcomes differed markedly. Among TSC-affected children, 16 of 22 (72.7%) developed epilepsy and 8 of 22 (36.4%) had neurodevelopmental delay. TSC-related findings are detailed in Table 4. All 15 children with isolated CR developed normally with no TSC manifestations.

**Table 4:** Clinical manifestations and incidence in the CR with the TSC group (*n* = 22).

Manifestation	*n* (%)
Epilepsy	
Present	16 (72.7)
Absent	6 (27.3)
Neurodevelopmental disorders	
Present	8 (36.4)
Absent	14 (63.6)
Other organ abnormalities	
Hypopigmented skin macules	4 (18.2)
Retinal hamartoma	2 (9.1)
Retinal pigmentary loss	1 (4.5)
Eyelid hamartoma	1 (4.5)
Multiple renal cysts	5 (22.7)
Renal angiomyolipoma	1 (4.5)
Multiple bone sclerosis foci	1 (4.5)

#### Fibroma

Five fibromas were diagnosed at a mean gestational age of 29.14 ± 5.57 weeks. The mean tumor volume was 23.29 ± 23.41 cm^3^ (range: 3.36–61.96 cm^3^), which was significantly larger than that of CR, myxoma, and unclassified tumors (*p* < 0.001 for all comparisons). All fibromas were solitary, with 80% (4/5) located in the left ventricular wall. On ultrasound images, fibromas appeared as well-circumscribed, homogeneous or heterogeneous, medium- to slightly hyperechoic masses with broad bases. One case demonstrated internal calcification (Figures 2C,D). Three cases developed complications, including pericardial effusion, premature ventricular contractions, and mitral regurgitation. One pregnancy was terminated at the family’s request despite the absence of significant antenatal complications. The remaining four infants were live-born. Among these, three cases underwent surgical treatment due to postnatal cardiac dysfunction, abnormal heart rate, and progressive tumor enlargement over a 5-year period, with favorable outcomes. The remaining case showed no significant tumor change during a 2-year follow-up period and also had a good clinical outcome.

#### Myxoma

Three myxomas were diagnosed at a mean gestational age of 26.36 ± 4.07 weeks. All myxomas were solitary and located in the interatrial septum, left atrial wall, and mitral valve. The mean tumor volume was 0.48 ± 0.33 cm^3^ (range: 0.16–0.81 cm^3^). On ultrasound images, myxomas appeared as slightly hyperechoic lesions with loose internal echogenicity (Figure 2E). A characteristic feature was mobility of the tumor with cardiac motion. All three cases had mild pericardial effusion. One had heart failure, one had an increased cardiothoracic ratio, and one had supraventricular tachycardia. Two pregnancies were terminated; the remaining live-born infant underwent surgical resection with a good outcome.

#### Teratoma

A single case of intrapericardial teratoma was diagnosed at 29 + 1 weeks of gestation. The tumor arose from the right atrial appendage and protruded into the pericardial cavity. The tumor volume was 6.81 cm^3^, and the mass had a complex cystic-solid appearance with relatively abundant internal vascularity originating from the right coronary artery (Figures 2F,G). Complications included massive pericardial effusion and a large right coronary artery-to-right atrial fistula. Due to the large tumor size and severe complications, the family elected to terminate the pregnancy.

#### Hemangioma

A right atrial hemangioma was detected at 39 + 3 weeks of gestation, with a volume of 3.63 cm^3^. Ultrasound revealed a slightly hyperechoic lesion with internal small anechoic areas resembling a “spongiform” pattern (Figures 2H,I). Notably, five prior ultrasound examinations (including the second-trimester anatomy scan) were negative. The neonate was delivered at term. The tumor did not regress spontaneously. Surgical resection was performed at 45 days of age, with a good outcome and no recurrence at the 1-year follow-up.

#### Unclassified tumors

There were five solitary tumors that were small in size and lacked distinctive imaging features and were therefore categorized as unclassified. The mean gestational age at diagnosis was 31.88 ± 5.99 weeks, and the mean volume was 0.34 ± 0.36 cm^3^. Echocardiographically, all five tumors were well-circumscribed, non-infiltrative, and predominantly hyperechoic or isoechoic relative to the adjacent myocardium, with no detectable internal vascularity on color Doppler. None of the tumors caused intracavitary obstruction, valvular dysfunction, or impaired ventricular contractility. On serial prenatal ultrasound examinations performed every 2–4 weeks and during regular postnatal follow-up (median follow-up: 48 months; range: 22–49 months), all tumors remained unchanged in size and morphology, and no infant developed new symptoms or cardiac dysfunction. None required surgical intervention, and all had favorable outcomes.

## Discussion

Fetal cardiac tumors are a group of rare and heterogeneous diseases with diverse pathological types and markedly different clinical prognoses, making accurate prenatal diagnosis and individualized prognostic assessment crucial. The overall detection rate of FCTs in this cohort was 0.062%, consistent with previous literature (1, 2). CR predominated (81.5%), followed by fibroma (6.2%) and myxoma (3.7%), with only one case of teratoma (1.2%). This differs from most reports in which teratoma is the second most common type (19), possibly reflecting regional or population differences. However, given that our institution is a tertiary referral center for fetal congenital heart disease, referral bias should be considered when interpreting both the detection rate and the pathological spectrum, as more complex or symptomatic cases may be disproportionately referred to our center. Additionally, this study reports an extremely rare case of fetal cardiac hemangioma. In this single-center cohort of 81 fetal cardiac tumors, which represents one of the larger series reported to date, several findings are worth highlighting for clinical practice.

### Considerable diagnostic value of echocardiography in FCTs

Echocardiography is the preferred tool for the diagnosis of FCTs. Different types of FCTs exhibit variations in location, number, size, and echocardiographic features (19). This study demonstrates that echocardiography has a high diagnostic accuracy for FCTs, with different tumor types presenting with certain characteristic features. For instance, CR typically appears as hyperechoic nodules protruding into the cardiac cavity and is often multiple. Fibromas usually present as medium-echoic, large tumors with a broad base and possible calcification. Myxomas tend to have a loose internal echotexture, often attaching to the atria or valves and moving with the cardiac cycle. Hemangiomas may show a “spongiform” echo pattern internally, while teratomas commonly manifest as cystic-solid masses within the pericardial cavity accompanied by pericardial effusion. However, these features are not entirely specific, and overlapping imaging appearances are common. In addition, it should be noted that the majority of tumors were detected in the second or third trimester, with more than half (50.6%) identified after 30 weeks of gestation. Indeed, one hemangioma was not detected until 39 weeks of gestation. These findings suggest that careful scanning of cardiac structures should be continued even into late gestation to avoid missed diagnoses.

### Tumor multiplicity, rather than size, shows stronger association with TSC in fetal CR

Identifying prenatal features of CR that predict TSC is clinically important (20, 21). Some studies have associated larger tumor volume with TSC (2), while others have emphasized the predictive value of tumor multiplicity (13, 22). In our cohort, multiple CRs were strongly associated with TSC: TSC was identified in 78.7% of fetuses with multiple lesions, compared with only 38.5% of those with solitary lesions (*p* = 0.013). This finding supports multiplicity as a key predictor of TSC. In contrast, maximum tumor volume showed no significant difference between TSC-associated and isolated cases (*p* = 0.646). To account for potential intercorrelations among tumor number, tumor size, and gestational age, we conducted an ANCOVA adjusted for gestational age at diagnosis and tumor number. Following adjustment for these two confounders, the intergroup difference in tumor volume remained non-significant, supporting the robustness of our primary findings. This finding differs from the prior report by Behram et al. (2), likely due to disparities in sample sizes. Our study included 60 CR cases with known TSC status, which was more than three times larger than their series of 18 cases. Notably, however, a solitary lesion does not exclude TSC, as nearly 40% of such cases in our cohort were TSC-related. We recommend that fetuses with CR undergo fetal brain MRI and genetic testing.

### The incremental value of fetal brain MRI

Prenatal detection of TSC-related brain lesions by ultrasound alone is known to be limited (23, 24). Subependymal nodules, a hallmark of TSC, are often too small and isoechoic to be visualized on sonography regardless of operator skill or equipment quality (23). In our cohort, ultrasound alone detected intracranial lesions in only 46.2% of TSC cases. Adding fetal brain MRI increased the detection rate to 69.2%. MRI, with its superior soft-tissue contrast, readily identifies subependymal nodules as small protrusions into the lateral ventricles (25).

However, a negative MRI does not exclude TSC. Jin et al. (10) reported that none of the 17 fetuses with negative prenatal MRI had pathogenic TSC1 or TSC2 variants. In contrast, 30.8% of TSC cases in our cohort had negative prenatal MRI. These fetuses either developed intracranial lesions postnatally or had TSC confirmed by genetic testing without detectable brain lesions. Thus, MRI complements but does not replace genetic testing. For families who decline genetic testing, a negative MRI provides some reassurance but not definitive exclusion.

### Factors associated with tumor-related complications

Previous studies have shown that FCT-related complications are closely associated with tumor size and location (14, 26). In our study, 25 fetuses (30.9%) developed complications, predominantly including pericardial effusion, valve regurgitation, outflow tract obstruction, arrhythmia, and an increased cardiothoracic ratio. Complication occurrence was not associated with tumor multiplicity (*p* = 0.195) but was significantly associated with tumor volume (*p* < 0.001). Additionally, all six outflow tract obstruction cases resulted from tumors located in the outflow tract region. Thus, tumor size and location are key factors in assessing hemodynamic impact and should be closely monitored on prenatal ultrasound.

Arrhythmia is a common complication of FCTs. Previous reports indicate that 13–37% of CR cases are associated with arrhythmias, with the types varying according to tumor location (27, 28). However, only three CR fetuses (4.5%) in our study had arrhythmias, a lower rate possibly due to the lack of routine fetal magnetocardiography. Fibromas involving the ventricular conduction system can induce potentially fatal ventricular arrhythmias (29), and hemangiomas have been reported to be associated with arrhythmias (30). In our study, one fibroma extensively involving the interventricular septum was associated with premature ventricular contractions, and one myxoma case was associated with paroxysmal supraventricular tachycardia, possibly related to direct invasion or mechanical irritation of the conduction system.

### Clinical trends and management

The prognosis of FCTs is largely determined by tumor histology and the presence of associated genetic syndromes. In our series, 59.5% of CRs regressed spontaneously after birth, consistent with their generally benign course (31). Among TSC-associated cases, however, 72.7% developed epilepsy and 36.4% exhibited neurodevelopmental delay. These findings should be interpreted with caution, as four cases with incomplete postnatal follow-up were excluded, and five unclassified tumors (some of which might have been CR) were not included in the CR subgroup analysis; both factors may have influenced the observed regression rate. In addition, TSC prevalence may have been affected by differential follow-up compliance between TSC-affected and non-TSC families, and the neurodevelopmental rates may be inflated, as symptomatic children are more likely to remain under surveillance. Moreover, five TSC-affected infants had follow-up of less than 24 months, which may underestimate later-onset cognitive or behavioral impairments. Despite these caveats, the substantial burden of neurological morbidity in TSC-associated cases underscores that long-term prognosis is driven primarily by TSC-related central nervous system involvement rather than by the cardiac tumor itself. Accordingly, prenatal counseling for suspected CR should shift its focus from tumor-related risks to TSC screening and neurological prognostication.

For fibromas, management is dictated by hemodynamic impact. Fibromas may remain stable or exhibit slow growth after birth (32), and in cases with extensive tumor involvement and severe complications, surgical resection or even cardiac transplantation may be required (32, 33). Among the four live-born cases, three required surgical resection due to progressive enlargement or refractory arrhythmia, with favorable postoperative outcomes. The remaining case remained stable, and for asymptomatic patients, an observation and waiting strategy could be adopted. The single hemangioma in this cohort exhibited rapid late-gestation growth (undetected at 34 weeks but diagnosed at 39 weeks) and did not regress postnatally, necessitating resection at day 45 with a good mid-term outcome. This pattern contrasts with the typical regression observed in CRs and suggests that hemangiomas may require active postnatal surveillance.

The only live-born myxoma case underwent surgical resection due to hemodynamic compromise, with no recurrence at 2 years. The intrapericardial teratoma presented with massive effusion and a coronary fistula, leading to pregnancy termination, reflecting its historically poor prognosis. Notably, all five unclassified tumors remained small, stable, and free of complications, and none required postnatal intervention. This subset may represent an indolent or benign entity for which conservative management is appropriate.

**Figure 3:**
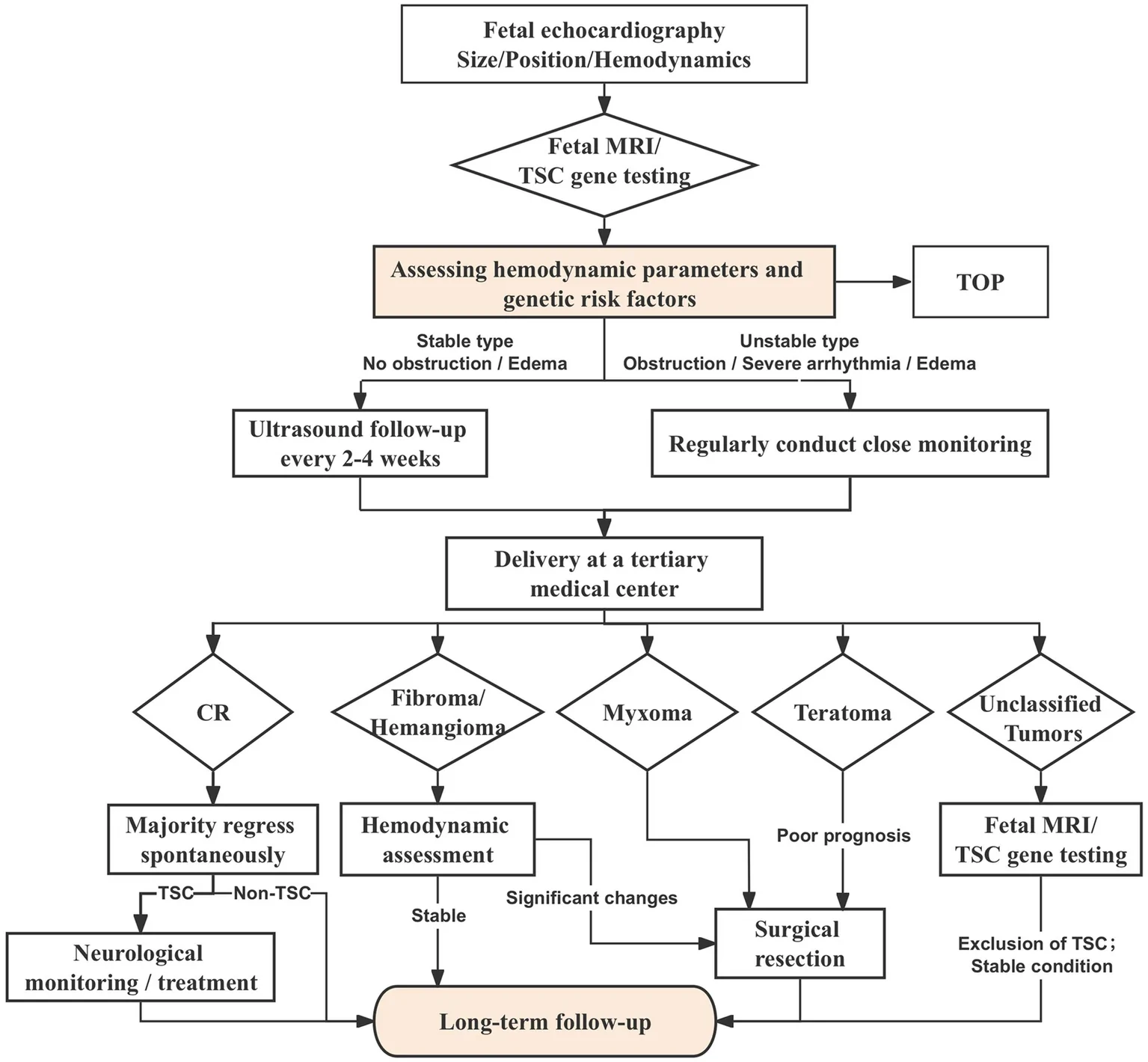
Flowchart of prenatal and postnatal management for FCTs.

Collectively, these findings support a pathological subtype-guided management strategy. CR warrants TSC evaluation and neurological follow-up. Fibromas and myxomas should be considered for elective resection if they are hemodynamically significant; asymptomatic cases can be observed. Hemangiomas may grow rapidly during late gestation, so close monitoring is necessary. Teratomas often have a poor prognosis. We summarized the prenatal and postnatal management flowchart for FCTs (Figure 3).

## Limitations

The limitations of this study include the following: (1) its single-center retrospective design, which may introduce selection bias; (2) five TSC-affected infants had follow-up of less than 24 months, whereas longer follow-up is needed to detect learning disabilities and behavioral problems that may emerge later; and (3) the small numbers of rare tumor types limit the generalizability of our conclusions.

## Conclusion

CR is the most common type of fetal cardiac tumor. The presence of multiple lesions, rather than the size of the tumor, is an important predictive indicator for TSC. Combining fetal brain MRI and ultrasound improves prenatal TSC detection. We recommend combined ultrasound, MRI, and genetic testing for all suspected CR, with counseling addressing both cardiac and neurological outcomes. Non-CR tumors are rare but require pathology-specific management strategies. Hemodynamic compromise depends on tumor volume and location.

## Data Availability

The original contributions presented in the study are included in the article/supplementary material, further inquiries can be directed to the corresponding authors.
